# Effect of 3 wt% Cu on the Microstructure and Hardness of a Ti-10Ta-1.6Zr Alloy

**DOI:** 10.3390/ma18133163

**Published:** 2025-07-03

**Authors:** Nobom G. Hashe, Lee Fowler, Susanne Norgren, Lesley A. Cornish, Lesley H. Chown, William E. Goosen, Johan E. Westraadt, Nomsombuluko D. E. Hadebe, Caroline Öhman-Mägi

**Affiliations:** 1 Department of Physics, Nelson Mandela University (NMU), Port Elizabeth 6031, South Africa; 2 Department of Materials Science and Engineering, Uppsala University, 75121 Uppsala, Sweden; fowler.lee8@gmail.com (L.F.); susanne.m.norgren@sandvik.com (S.N.); caroline.ohman@angstrom.uu.se (C.Ö.-M.); 3 School of Chemical and Metallurgical Engineering, Hosted by the University of the Witwatersrand, Johannesburg 2000, South Africa; lesley.cornish@wits.ac.za (L.A.C.); chownlesley@gmail.com (L.H.C.); 4 DSI-NRF Centre of Excellence in Strong Materials, Hosted by the University of the Witwatersrand, Johannesburg 2050, South Africa; 5 Centre for High Resolution Transmission Electron Microscopy, Nelson Mandela University (NMU), Gquberha 6001, South Africa; william.goosen@mandela.ac.za (W.E.G.);; 6 Mintek, Advanced Materials Division, Randburg 2194, South Africa

**Keywords:** microstructure, hardness, titanium, tantalum, copper

## Abstract

Alloys of Ti-10Ta-1.6Zr (wt%) with and without 3 wt% Cu made by arc-melting, heat-treated in two stages and quenched to have α + β microstructures were studied. These alloys were studied for potential replacement of Ti-6Al-4V alloys because Ta and Zr are more biocompatible than Al and V, and copper was added for potential antimicrobial properties. The heat-treated samples were investigated by SEM-EDX, transmission Kikuchi diffraction (TKD) and XRD. When studied at a higher magnification, the heat-treated alloys revealed a bi-lamellar microstructure, consisting of broad α lamellae and β transformed to fine α′ lamellae with various orientations. The fraction β transformed to fine α′ lamellae was higher in the alloy with Cu than that without Cu. Furthermore, copper was found to lower the solubility of tantalum in the β. The hardest alloy was the heat-treated alloy containing Cu, albeit with a wide standard deviation, probably due to the high fraction of martensitically transformed β.

## 1. Introduction

Biomaterials are used in different parts of the human body, e.g., as artificial valves in the heart, and stents in blood vessels, as well as shoulder, knee, hip, elbow, ear and dental implants [1,2,3]. It has been customary to utilise Co-Cr, and 316L austenitic steel as metallic orthopaedic implants because of their mechanical properties. However, these materials demonstrate larger Young’s moduli than human bone and can be cytotoxic [4]. Nickel–titanium alloys are used as dental wires, stents, etc., because of their excellent corrosion-resistant properties, adequate mechanical strength and shape-memory effect. However, they have a risk of metal allergy because of leaking nickel ions in aqueous conditions [5]. Reviewing toxicological risks of Co-Cr alloys, Grosgogeat et al. [6] stated that most in vitro studies showed Co-Cr alloys to have better cytocompatibility than Ni alloys. Titanium and its alloys are used in orthopaedic and dental applications because of their excellent biocompatibility, high corrosion resistance, low density, high strength/weight ratio, high ductility and low thermal conductivity [7,8,9,10]. Patel and Gohil [3] showed that although the elastic moduli of Ti-alloys are lower than for Co-Cr alloys and stainless steels, they are still higher than for cortical bone. The most used titanium alloy is Ti-6Al-4V, due to its higher mechanical strength. However, aluminium and vanadium have been found to cause adverse reactions and their presence in the body is therefore unwanted [11]. Attention has been given to titanium alloys with the addition of niobium (Nb), tantalum (Ta) and zirconium (Zr), i.e., “TNTZ” alloys, due to their biocompatibility and comparable strength to that of Ti-6Al-4V [12,13,14,15,16,17,18,19,20]. Niobium, tantalum and zirconium belong to a group of metals that form highly stable oxide layers, which act as barriers to any corrosion medium, giving the alloy a high corrosion resistance. Tantalum and niobium are also β-stabilisers, while zirconium acts as a neutral element [17,21]. Yang et al. [15] investigated the effect of Nb on Ti-*x*Nb-8Sn alloys (where *x* = 16, 20, 24, 28, 36 wt%), finding that varying Nb content changed the phases from (α″ + β) at 16 wt% to single-phase β at 36 wt%, i.e., increased Nb increased the volume fraction of β [15]. The tensile properties of the Ti-*x*Nb-8Sn alloys were also strongly affected by the Nb content [15]. Dercz et al. [16] corroborated Nb as a β-stabiliser in Ti-Ta-Nb-Zr alloys. Furthermore, the study found that increased zirconium content increased the grain size [16]. Ti-30Ta-10Zr-20Nb (wt%) and Ti-30Ta-20Zr-10Nb (wt%) alloys with higher Zr and Nb contents had higher microhardness and improved corrosion resistance [16]. Dobri et al. [17] showed that Ta increased the β volume fraction and reduced the elastic modulus to be closer to that of human bone for Ti-*x*Ta-9Nb-8Zr-2Ag (where *x* = 10–20% Ta) alloys.

Another concern is that implants are undesirably affected by bacterial growth that causes infections and inflammation in the surrounding tissues. Prosthetic implant infections are associated with the formation of persistent biofilms on the surface of the implants. A common way of treating this type of infection has been antibiotics, although with the growing problem of antibiotic resistance [22], new solutions are needed. Antibacterial alloys could potentially solve at least part of the problem. Recently, attention has been given to copper-containing alloys, due to copper’s antibacterial properties [23,24,25,26,27,28,29,30,31,32,33]. It has been suggested that 3 wt% copper is the minimum amount needed for antibacterial effectiveness [27]. Mainly binary Ti-Cu alloys have been investigated, with some exceptions. Ren et al. [28] and Zhuang et al. [29] studied the addition of Cu to Ti-6Al-4V and found a promising antibacterial effect. However, the microstructure of the alloys and the effect of heat treatment were not investigated. Fowler et al. [23] investigated the effect of 0–10 wt% copper additions to a Ti-10.1Ta-1.7Nb-1.6Zr (TTNZ) alloy. The alloys with 3 wt% Cu and more were three-phase alloys, containing α, Ti_2_Cu and a bright-contrast phase that could not be indexed (hypothesised to be a martensitic phase). Most of the Cu precipitates had Cu/Ti ratios corresponding to Ti_2_Cu, although a few corresponded to Ti_3_Cu. Thus, a thorough study of binary Ti-Cu alloys to clarify the phase diagram was carried out, recognising Ti_2_Cu as stable and Ti_3_Cu as metastable [34]. In the study of Fowler et al. [23], it was also found that a higher Cu content gave a coarser structure. Furthermore, the hardness significantly increased with an increase in Cu content up to 3 wt%, while a further increase did not have an effect on the hardness. It was hypothesised that this was due to the saturation limit of solid solution alloying being reached. Wang et al. [30] added Cu to Co-Cr-W-Ni, finding decreased corrosion resistance and slightly decreased microhardness. The alloys showed good cell viability and Cu additions did not cause cell toxicity [30]. However, adding Cu to Co-Cr-W increased the grain size, reduced the mechanical properties, and increased corrosion current density, even though there was no cytotoxicity [31]. Duan et al. [32] synthesised Co-Cr-Mo with different Cu amounts, finding that increased Cu content reduced microhardness, while the yield strength and ultimate tensile strength increased. They also found that Cu stabilised the FCC Co phase. The effect of Cu on 316L stainless steel after solution and ageing treatment was studied by Xi et al. [33], finding different mechanical properties from the different heat treatments. The addition of Cu and ageing treatment were important to improve the antibacterial properties of steel [33]. Chong et al. [35] investigated the microstructure evolution in Ti-6Al-4V and found that annealing between 800 °C and 960 °C caused a bi-lamellar microstructure, i.e., coarse primary α plates and the transformed β comprised fine secondary α and retained β. This type of structure had previously been proposed by Lütjering [36] and might explain the inconclusive microstructures found in the TTNZ-Cu alloys [23].

The aim of this study was to further investigate the impact of Cu on the microstructure of multicomponent titanium alloys. To simplify the alloy, only Ta was added as a β-stabiliser to give an α + β alloy, and the effect of a 3 wt% Cu addition to the Ti-10.1Ta-1.6Zr alloy was compared to an alloy without the Cu addition for the same heat treatments.

## 2. Modelling and Experimental Procedures

### 2.1. Computational Modelling of Alloys

The Thermo-Calc software (v. 2021, Thermo-Calc Software AB, Stockholm, Sweden) was used prior to the experiments to predict the likely phases and their compositions. The Scientific Group Thermodata Europe solutions’ database version 5 (SSOL5 database) [37] was used entirely for the Ti-Ta-Zr system, and for the Ti-Ta-Zr-Cu system, the Cu-Ta binary by Lin et al. [38] was appended. The SSOL5 database includes the Cu-Ti binary assessed by Kumar et al. [39], which had good agreement with experimental data near the terminal elements, but less good agreement elsewhere [34]. An alternative description of Cu-Ti by Canale and Servant [40] includes a stable Ti_3_Cu phase, which was not observed in earlier work on a similar alloy [24]; so, the description of Kumar et al. [39] was preferred. The Cu-Ti, Ta-Ti, Ti-Zr, Ta-Zr and Cu-Zr binaries were all included in the SSOL5 database, as well as the Ta-Ti-Zr and Cu-Ti-Zr ternaries, but Cu-Ta-Ti and Cu-Ta-Zr were missing. Thus, the predictions can be used with good accuracy for the Ti-Ta-Zr system but only as a guide for the Ti-Ta-Zr-Cu system.

### 2.2. Alloy Preparation

To produce the Ti-Ta-Zr-Cu alloys, 3 wt% of 99.9999 wt% pure copper rods (365327-21.5G, Sigma Aldrich, MO, USA) were mixed with the pre-alloyed TTZ alloy (Sandvik AB, Stockholm, Sweden). The TTZ alloy was produced in the same way as described by Stenlund et al. [20].

**Figure 1:**
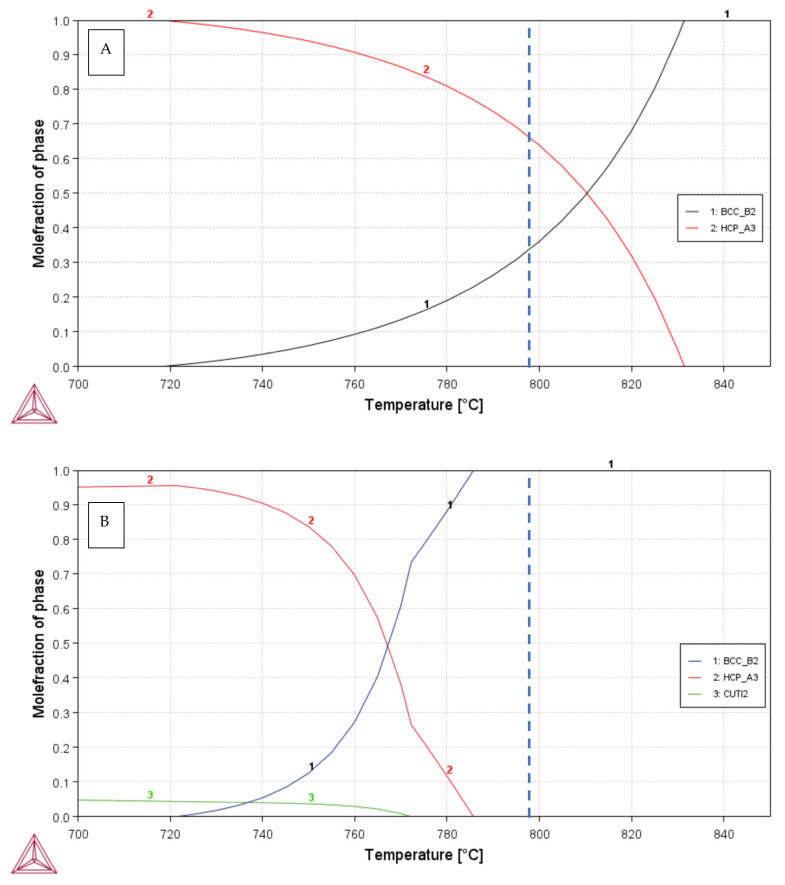
Mole fractions of phases as a function of temperature, α = HCP_A3 in red, β = BCC_ B2 in blue and Ti_2_Cu = CUTI2 in green for (**A**) TTZ alloy and (**B**) TTZ-Cu alloy. The dashed line indicates the heat treatment temperature of 798 °C.

The TTZ alloys with and without 3 wt% Cu were re-melted five times in an arc-furnace using a W-filament (Series 5 Bell Jar, Centorr Vacuum industries, Nashua, NH, USA). The alloy buttons were turned over between the melting cycles to achieve a higher degree of homogenisation. Two melts of each alloy were carried out: one melt was left as-cast (AC) and the other was heat-treated (HT) in a vacuumed and sealed quartz-ampoule at 1.333 mbar, which was placed in a conventional tube furnace. The heat treatment comprised two sequential steps: 980 °C for 10 h, followed by 798 °C for 6 h, and finally a rapid salt-brine water quench. The annealing temperatures were chosen according to the estimated molar phase fractions of α (HCP) and β (BCC) for the TTZ alloy shown in Figure 1. The first heat treatment temperature (980 °C) was above the β-transus for both alloys, as shown in Figure 1, and the second (798 °C) was in the α region for the TTZ but in the α + β region for the TTZ-Cu alloy according to the Thermo-Calc calculations carried out.

### 2.3. Sample Preparation

The as-cast (AC) and heat-treated (HT) samples were cut and then ground to create flat surfaces. They were then polished following the standard method for polishing Ti-based alloys. The samples were polished using Aka-Daran and Mag-Napal cloths and suspensions from 6 to 3 µm and 1 to 0.25 µm, respectively. The final polish was made using a Mag-Chemal cloth and a suspension of oxide polishing suspension (50 nm SiO_2_ + hydrogen peroxide).

### 2.4. XRD and Microscopy Analysis

The samples were analysed in a JEOL JSM-7001F FEG scanning electron microscope (SEM) (JEOL, Tokyo, Japan) at 15 kV, using secondary electron (SE), backscattered electron (BSE) and energy-dispersive X-ray spectrometer (EDS) detectors. For crystallographic investigations, the heat-treated alloys were studied further after preparation of focused ion beam (FIB) samples, analysis with STEM-EDS and transmission Kikuchi diffraction (TKD).

The polished samples were also analysed using XRD: a Bruker D2 with Cu radiation with a Lynxeye detector (Bruker, Billerica, MA, USA). The scan ranged from 30° to 110° 2θ at 0.02° scan steps.

### 2.5. Image Analysis

The area fractions of phases were calculated from the SEM images using ImageJ software 2.9.0/1.53t. The contrast between the bright- and dark-contrast phases was high and hence the threshold was set using the automatic feature included in the software.

### 2.6. Hardness Measurements

Macro Vickers hardness tests were carried out randomly on the polished samples using an Emcotest Duravision Vickers hardness machine (EMCO-TEST, Kuch-Salzburg, Austria), under a 10 kg load, according to ASTM Standard E92-17 [41]. The indentations were about 3–5 mm apart and averages of six measurements were obtained for each sample and condition.

## 3. Results

The Thermo-Calc calculations showed that the first heat treatment temperature 980 °C was well above the β-transus for both alloys. Thus, heat treatment would generate large β grains, which were observed in the microstructures. The second heat treatment temperature was 798 °C, which Thermo-Calc indicated as the α + β region with 65 vol.% α and 35 vol.% β for TTZ, while the TTZ-Cu alloy was still in the β field, as shown in Figure 1. However, as already stated, the calculations for this alloy were a guide only, as two ternary systems (Cu-Ta-Ti and Cu-Ta-Zr) were missing. The calculated composition of β in TTZ at 798 °C was 17.2 wt% Ta and 1.8 wt% Zr, and for α, it was 6.0 wt% Ta and 1.5 wt% Zr. Thermo-Calc predicted the Ti_2_Cu phase to precipitate in the solid state in the TTZ-Cu alloy.

**Figure 2:**
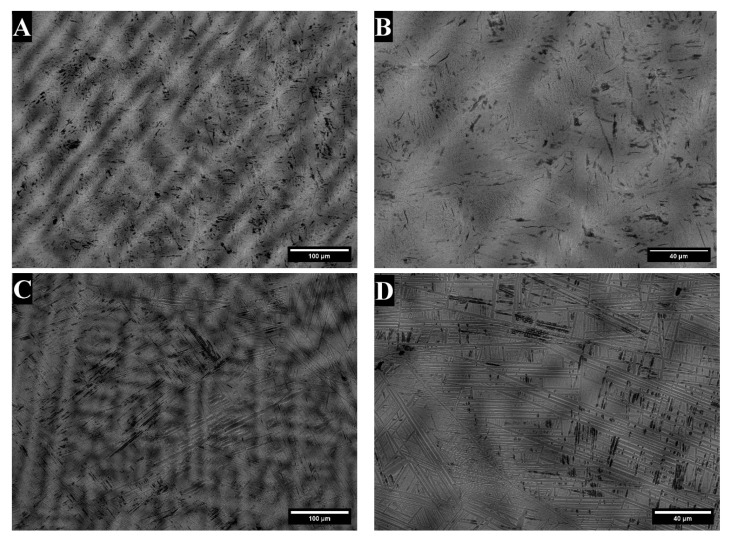
SEM-BSE images of as-cast alloys: (**A**,**B**) TTZ, and (**C**,**D**) TTZ-Cu.

The microstructures of the as-cast samples are given in Figure 2. Dendrites were visible in both the TTZ and TTZ-Cu alloys (Figure 2A,C) after casting. At a higher magnification (Figure 2B,D), the microstructures were lamellar.

**Figure 3:**
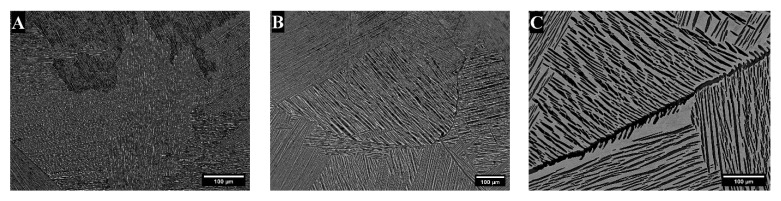
Representative SEM-BSE images of (**A**) TTZ-HT that had overall dark-contrast areas only, (**B**) TTZ-Cu-HT showing overall dark-contrast areas and (**C**) overall bright-contrast areas of the TTZ-Cu-HT.

**Figure 4:**
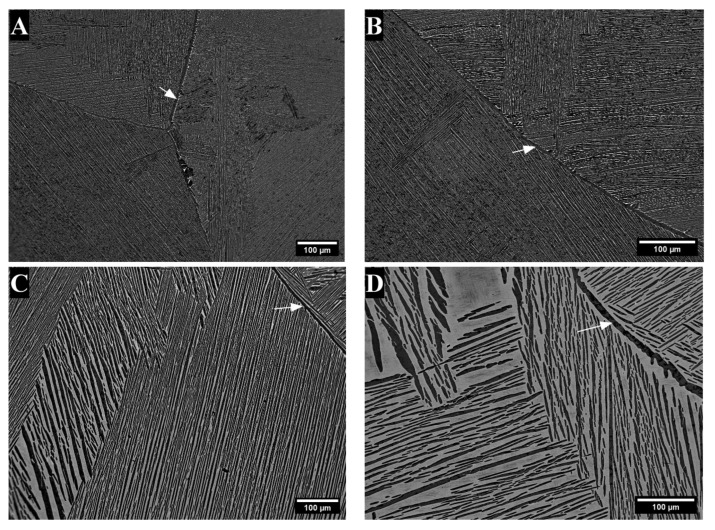
SEM-BSE images of (**A**,**B**), TTZ-HT showing only dark-contrast areas, and (**C**), TTZ-Cu-HT showing dark- and bright-contrast (**D**) areas compared to TTZ-HT. Arrows indicate prior β grain boundaries.

**Figure 5:**
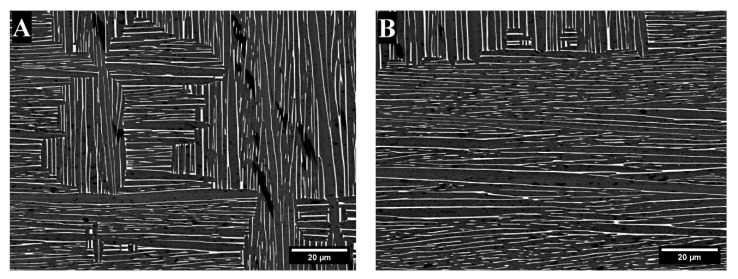

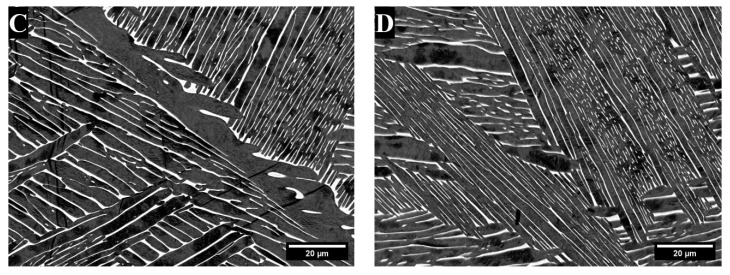
SEM-BSE images at higher magnification showing (**A**,**B**) TTZ-HT, and (**C**,**D**), TTZ-Cu-HT.

After heat treatment, the microstructures were lamellar, with traces of the large prior β grains. There were areas with overall dark-contrast areas in the TTZ-HT alloy (Figure 3A), while the TTZ-Cu-HT alloy had both dark and bright-contrast areas (Figure 3B and Figure 3C, respectively). The overall dark- and bright-contrast areas both consisted of bright- and dark-contrast phases, and were more clearly observed at higher magnification (Figure 4 and Figure 5).

The area fractions of phases were similar in the dark-contrast areas for both alloys (~20% bright-contrast phase), whereas the bright-contrast areas of the TTZ-Cu-HT had almost three times the amount (~58%) of the bright-contrast phase (Table 1 and Figure 3).

Figure 4 and Figure 5 give the microstructures of TTZ-HT and TTZ-Cu-HT at two different magnifications, revealing that the overall dark and bright-contrast areas consisted of bright- and dark-contrast phases. The TTZ-HT microstructure comprised an α phase that formed at the prior β grain boundaries and fine lamellae within the prior β grains. The TTZ-Cu-HT alloy was similar, but with more of the bright-contrast phase than TTZ-HT (Figure 4). In addition, the microstructure of TTZ-Cu-HT was coarser than that of TTZ-HT, indicating that the Cu addition lowered the β-transus temperature.

**Figure 6:**
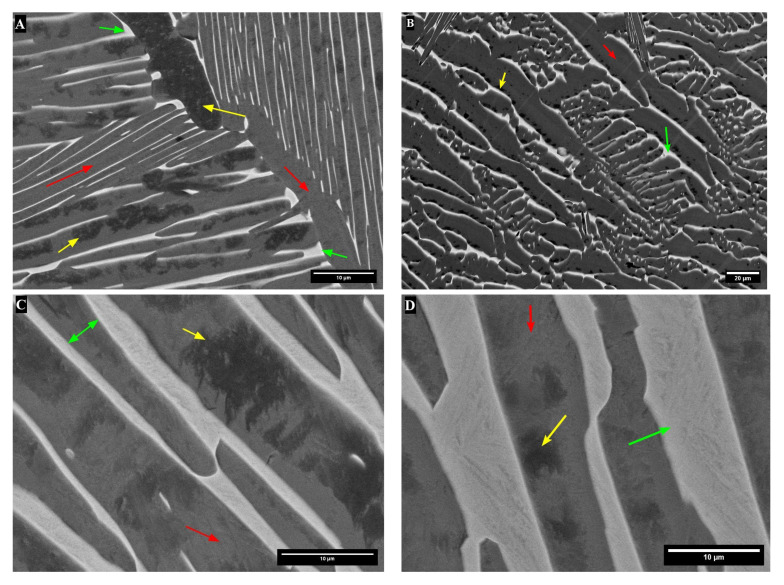
SEM-EDX micrographs showing where measurements were taken: (**A**,**B**) TTZ-HT and (**C**,**D**) TTZ-Cu-HT alloys, where yellow arrows show of the dark-contrast phase, red arrows show the medium-contrast phase and green arrows show the bright-contrast phase.

These higher resolution images (Figure 5) revealed that the dark-contrast phase had two different contrasts, herein called medium- and dark-contrast phases. EDX measurements were taken on all three contrasts, i.e., bright, medium- and dark-contrast phases. Figure 6 indicates the position of the individual measurements. Table 2 and Table 3 give the average composition of each phase in wt% with corresponding average compositions in at% (Table 4 and Table 5). The EDX analyses from the medium- and dark-contrast phases had the same compositions. The bright-contrast phase had, as expected, higher Ta and Ta + Cu contents than the darker contrast phase for TTZ-HT and TTZ-Cu-HT. The average composition of the bright-contrast phase (Table 2) in TTZ-HT was 20.2 wt% Ta, which is in fair agreement with the calculations that gave 17.2 wt% Ta in the β phase. In the bright-contrast phase in the TTZ-Cu-HT, the Ta content was only 14.5 wt%, while the Cu was 2.7 wt%, giving a total of 17.2 wt%, indicating that Cu reduces the solubility of Ta in the β phase. There were also traces of tungsten, which was contamination from the W-electrode in the arc furnace.

**Figure 7:**
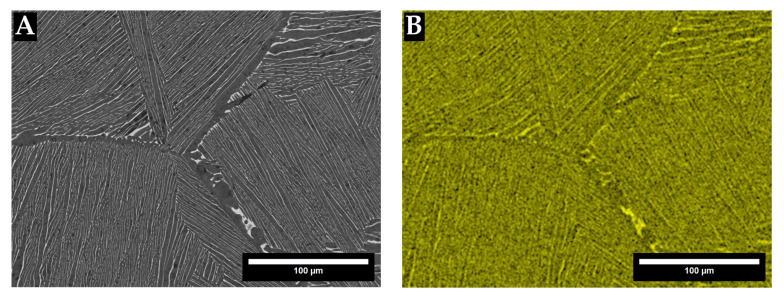
TTZ-HT alloy: (**A**) SEM-BSE image and (**B**) SEM-EDX mapping of Ta showing that the bright-contrast phase was rich in Ta.

**Figure 8:**
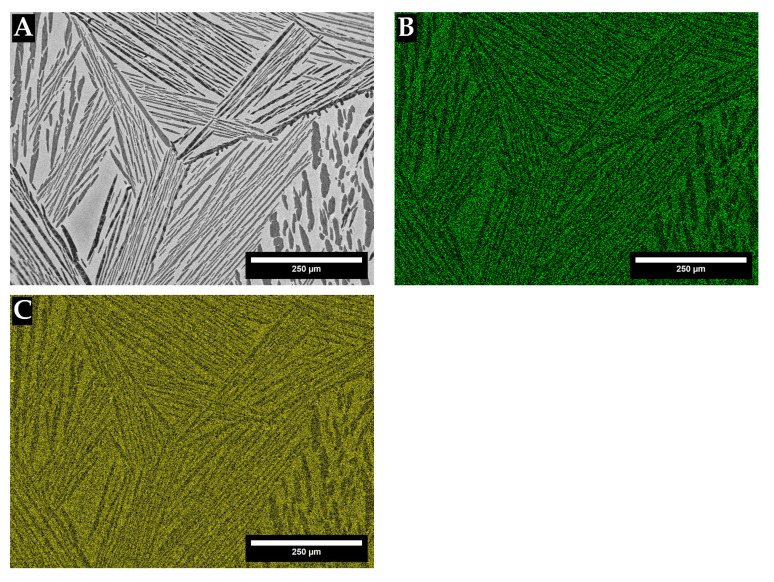
(**A**) SEM image of the TTZ-Cu-HT alloy and SEM-EDX mapping of (**B**) Cu and (**C**) Ta showing that the bright-contrast phase is rich in Ta and Cu.

Figure 7 and Figure 8 give SEM-EDX complementary mapping of the TTZ-HT and TTZ-Cu-HT alloys, confirming that the bright-contrast phase had higher Ta and Ta + Cu contents.

**Figure 9:**
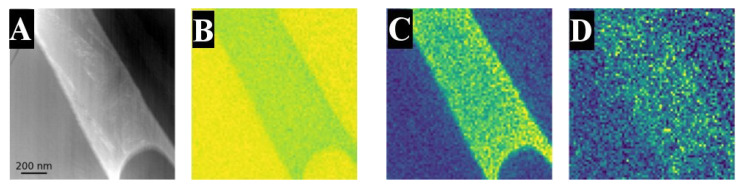

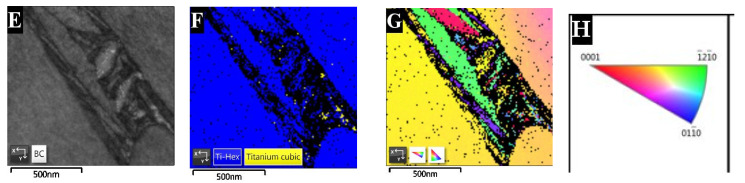
TKD and EDX results of TTZ-HT**:** (**A**) overview image of the area investigated with both techniques, (**B**–**D**) SEM-EDX maps of (**B**) Ti, (**C**) Ta and (**D**) Zr, (**E**) band contrast, (**F**) phase map, (**G**) IPF Z map of the same area and (**H**) its associated IPF Z colour key.

**Figure 10:**
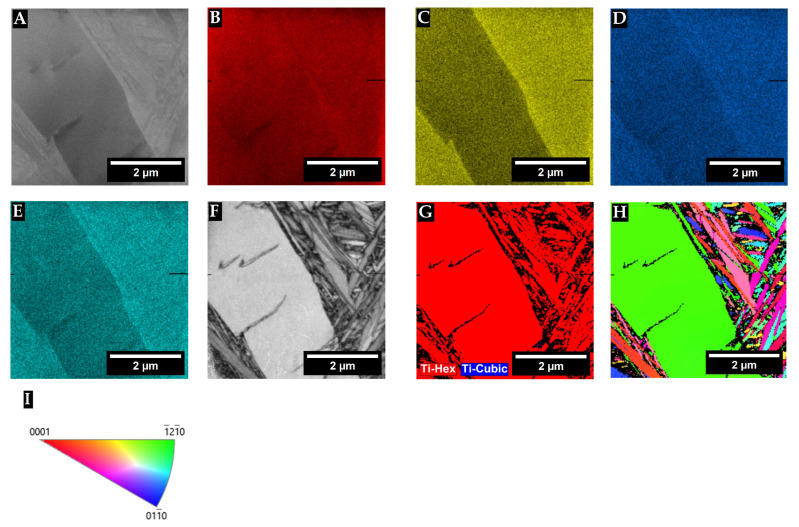
TKD and EDX results of TTZ-Cu-HT**:** (**A**) an overview image of the area investigated with both techniques, (**B**–**D**) SEM-EDX maps of (**B**) Ti, (**C**) Ta, (**D**) Zr and (**E**) Cu, (**F**) band contrast, (**G**) phase map, (**H**) IPF Z map of the same area and (**I**) its associated IPF Z colour key.

To further resolve the microstructure, the TTZ-HT and TTZ-Cu-HT samples were studied by TKD (Figure 9 and Figure 10) to reveal whether they had a substructure. Figure 9 and Figure 10 show the variation in the elemental composition in the bright-contrast phase, where (B), (C) and (D) are composition mappings of Ti, Ta, and Zr, respectively. The bright-contrast phase was rich in Ta and Ta + Cu and the composition was homogenous within it. The band contrast in Figure 9E shows evidence of a fine structure within the bright-contrast phase. Figure 9F,G indicates that it can partly be indexed as HCP, although with a higher Ta content than primary α and was thus assumed to be α′ formed martensitically during quenching. In Figure 10F, the TTZ-Cu-HT reveals more clearly that the bright-contrast phase had a very fine lamellar structure with random orientation (Figure 10H), which was not visible by SEM. It probably formed by martensite transformation of the retained β to α′ during the rapid cooling.

### 3.1. X-Ray Diffraction

**Figure 11:**
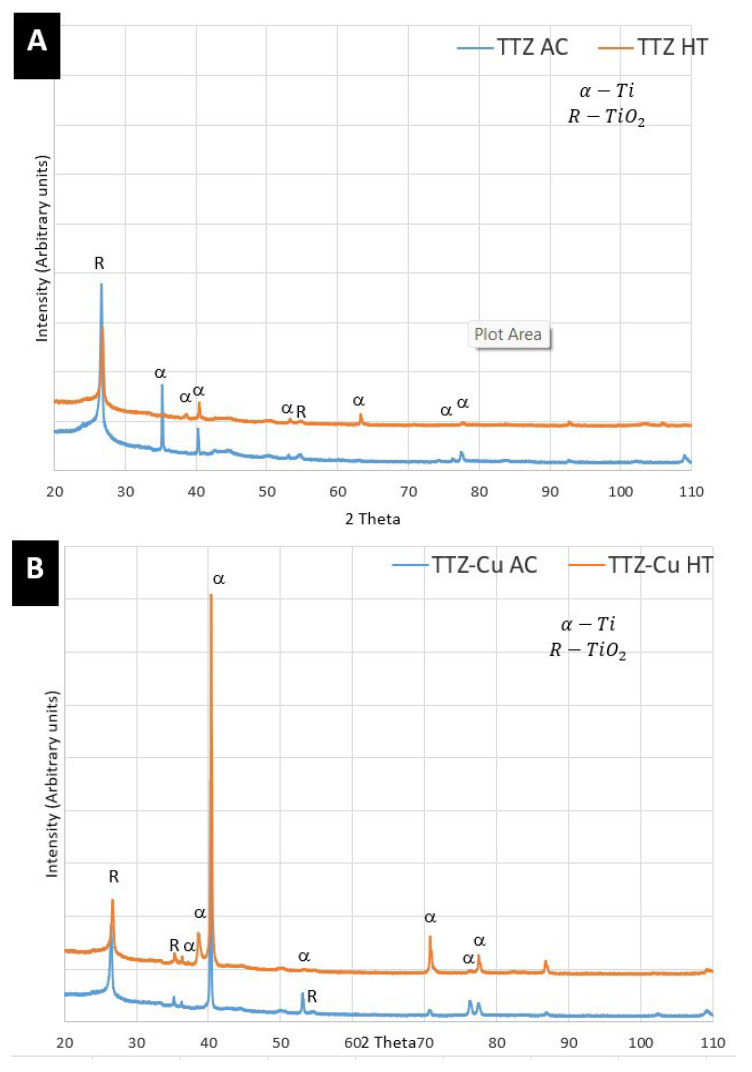
XRD diffractograms of (**A**) TTZ and (**B**) TTZ-Cu alloys indexed for α phase.

The XRD diffractograms for the alloys are given in Figure 11. Both as-cast and HT samples had patterns that matched that of Ta_0.03_-Ti_1.98_ by Maykuth et al. [42]. In the heat-treated alloys, the β phase was not detected using XRD. The peaks not corresponding to α at 2θ = ~26.7°, 2θ = ~35.3° and 2θ = ~55° could be the reflections from a surface oxide layer. Rutile has its three strongest intensities at approximately these 2θ values (JCPDS Card No. 21-1276 [43]). Titanium oxide was not observed in the microstructure by the analytical techniques used in this study, indicating that these peaks come from a surface oxide layer.

### 3.2. Hardness

**Figure 12:**
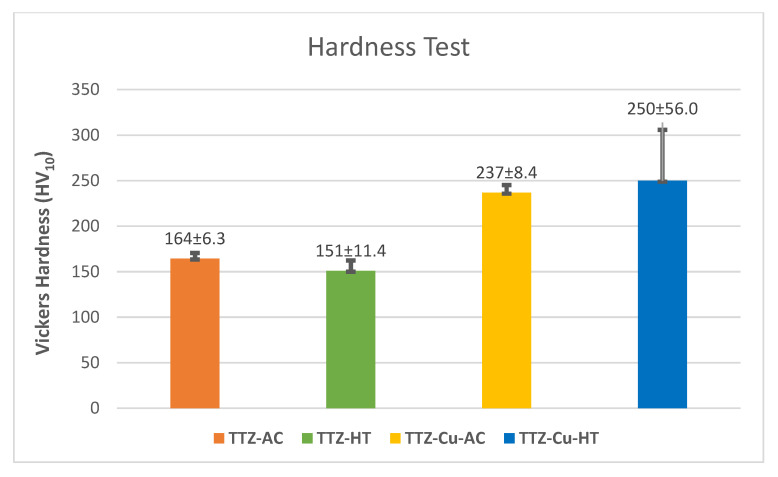
Hardness results of as-cast (AC) and heat-treated (HT) TTZ and TTZ-Cu alloys.

The hardness results are shown in Figure 12. The difference in hardness for each alloy between the AC and HT conditions was within the standard deviations. The addition of 3 wt% copper increased the hardness by an average of 70 HV_10_, although the standard deviation was higher for TTZ-Cu-HT.

## 4. Discussion

The TTZ and TTZ-Cu alloys were produced as as-cast and heat-treated samples, which were analysed and compared. The microstructure of both as-cast alloys retained the primary β dendrites (Figure 2A,B). At a higher magnification, α plates were observed in TTZ-Cu-AC (Figure 2D), although these were more difficult to discern in TTZ-AC (Figure 2C).

After heat treatment above the β-transus, the dendritic segregation pattern was homogenised and both alloys had lamellar microstructures. The major difference between the two heat-treated alloys was the higher fraction bright-contrast phase and the coarser lamellae in TTZ-Cu-HT (Figure 4 and Figure 5). This might be because the TTZ-Cu-HT alloy was heat-treated at a higher temperature relative to the β-transus temperature than that of TTZ-HT, as indicated by the thermodynamic calculations (Figure 1), which gave larger α lamellae. The bright-contrast phase in both heat-treated alloys (Figure 5) could be retained β. However, the β phase was not observed in either alloy (neither by XRD nor TKD); so, the bright-contrast phase was suspected to be retained β that transformed into α′ martensite. The prior β grain boundaries were visible in both alloys (indicated by arrows in Figure 4).

The SEM-EDX maps in Figure 7 and Figure 8 of the TTZ-HT and TTZ-Cu-HT display two phases in each alloy, the bright-contrast phase being richer in Ta or Ta + Cu than the dark-contrast phase. The EDX results gave no discernible composition difference between the medium- and dark-contrast phases in either of the alloys (Table 2 and Table 3, and Figure 7 and Figure 8), and it was assumed that the contrast difference was probably due to crystal orientation and/or twinning. This has also been observed by Chong et al. [35] in a Ti-6Al-4V alloy with similar structure.

The TTZ-Cu alloy was, according to the incomplete thermodynamic modelling, heat-treated first above the β-transus and thereafter in the β region. However, the microstructure was more in agreement with 798 °C being in the α + β region, demonstrating the need to include also the Cu-Ta-Ti and Cu-Ta-Zr ternaries in the modelling for it to be useful. Furthermore, it must be remembered that Thermo-Calc calculated the equilibrium phases, whereas the samples were quenched from the heat treatment temperature (798 °C) and martensitic transformation was not included in the calculations.

The microstructure consisted of the α phase that nucleated in the prior β grain boundaries and then grew into the grains. The proportion of bright-contrast phase corresponding to β that had transformed martensitically to α′ upon quenching was rather high in TTZ-Cu-HT and corresponded to ~60 area% (in some parts), according to image analysis (Table 3), which further indicated that 798 °C is in the α + β region. The martensite formed upon quenching in this β alloy that had a low amount of β-stabilising elements was α′ with the hcp structure [21]. An additional interesting observation (Figure 9D) was that Zr was enriched in the transformed β regions despite zirconium being known to be a neutral element [21].

Chong et al. [35] reported that a Ti-6Al-4V alloy heat-treated below the β-transus followed by quenching developed a microstructure where α first nucleated at the β grain boundaries and grew into the grains to form a lamellar structure, and then the remaining β transformed martensitically into a another α phase during quenching (α′). This gave a bi-lamellar microstructure composed of the broad original α plates and the transformed β comprised finer subsequent α′ with the same composition as the prior β phase [35]. The bi-lamellar microstructure has been reported as advantageous for good mechanical properties [36]. Comparing Chong et al.’s [35] with this study, in the TTZ-Cu-HT alloy, there were also α plates and the bright-contrast phase, composed of β transformed to fine α′ with the same composition as the primary β phase. Conversely, for the TTZ-HT alloy, there was very little retained β and the alloy was nearly all α. However, in the retained β, there was a substructure (Figure 9) and the composition was in agreement with primary β. Based on the results of Chong et al. [35] on Ti-6Al-4V, the bright-contrast phase is prior β that transformed into the subsequent α′ lamellar phase. The band contrast in Figure 9E and Figure 10F is evidence that TTZ-HT and TTZ-Cu-HT exhibited a bi-lamellar microstructure, which is in agreement with the results of Chong et al. [35]. Since the initial α and subsequent α′ are both hexagonal, it was difficult to differentiate them using TKD (Figure 9F and Figure 10G). Furthermore, Chong et al. [35] showed small amounts of retained β in the quenched Ti-6Al-4V that were only visible with TEM characterisation, a technique that was not used in the current study.

In the primary β (bright-contrast phase) in the TTZ-Cu-HT, the Ta content was, as mentioned above, only 14.5 wt%, while the Cu was 2.7 wt%, giving a total of 17.2 wt%, indicating that, as mentioned, Cu reduces the solubility of Ta in the β phase.

Contrary to Fowler et al. [23], who observed precipitates of Ti_2_Cu in a Ti-Nb-10Ta-1.6Zr-3Cu alloy, the heat-treated TTZ-Cu-HT alloy in this investigation had no discernible Ti_2_Cu. The quenching rate was most likely too rapid for the alloy to equilibrate, thus not allowing Ti_2_Cu to nucleate, possibly giving metastable microstructures, or simply that Ti_2_Cu is not stable under these conditions. This needs further investigations.

The XRD patterns clearly indexed α, with no clear β peaks. However, β peaks are difficult to distinguish from α peaks since they partly overlap. On the other hand, during quenching, a martensitic transformation of β to α′, also with an HCP structure, occurred.

The presence of the initial α was confirmed by XRD and SEM, and the subsequent α′ phase was confirmed by the TKD results (Figure 9F and Figure 10G). Since α′ has the same crystal structure as α with slightly different lattice parameters, it is difficult to distinguish between the two phases with XRD. The intensities of the peaks at around 2θ = 47° for all alloys were consistent with the highest intensity for the HCP structure (Figure 11A,B). However, this peak was more intense than the standard (Ta_0.03_Ti_1.98_ [42]) used. This could be caused by the current alloys consisting of more Ta than the standard and there being additional elements, such as Zr, and Cu, which were likely to shift the peak positions. In contradiction to the Ti_2_Cu observed by Fowler et al. [23], in the TNTZ-3%Cu also containing Nb, this phase was not observed in this study, using XRD, SEM and TKD. It is possible that the microstructure had small Ti_2_Cu precipitates, which were too small to resolve with the techniques used here; thus, TEM would be needed to resolve this and possibly also complementary heat treatments to investigate whether the phase is stable at these temperatures.

The hardness increase for both TTZ-Cu alloys can partially be attributed to the Cu, which would have given solid solution strengthening [23]; even though Cu is the least hard component, it would still have an effect because of the different atom sizes. Another contribution for TTZ-Cu-HT would be the bi-lamellar microstructure since it has been reported as advantageous for good mechanical properties [36]. Chong et al. [35] credited the high yield strength to heat treatment in the α + β intercritical annealing region. The increase in hardness for the TTZ-Cu alloys in this study was thus also due to the alloys being heat-treated in the α + β region. The larger spread in hardness values for the TTZ-Cu-HT alloy compared to TTZ-HT might also be attributed to the presence of bright- and dark-contrast areas. The overall hardness of the bright-contrast areas was expected to be higher, both due to a higher content of Ta and Cu as well as the bi-lamellar structure, while the hardness of the overall dark-contrast areas was comparable to that of TTZ-HT. It is known that Cu and Ta are β stabilisers [17,44]; accordingly, the addition of Cu increased the bright-contrast region, i.e., prior β. This bright-contrast region was where the bi-lamellar microstructure formed; hence, the increased hardness. The hardness of TTZ-Cu-HT (250 HV_10_) was similar to the hardness measured by Fowler et al. [23] in a TNTZ-3%Cu (238 HV_10_). The difference could be ascribed to the addition of niobium and heat treatments at different temperatures and durations. The image analysis has shown that there is a lower overall fraction of the bright-contrast phase. It has been speculated that the bright-contrast phase increases the hardness of the material due to the higher content of Ta. Therefore, it can be speculated that the lack of the bright-contrast phase plays a role in the decrease in the hardness of the heat-treated TTZ.

## 5. Conclusions

Alloys of Ti-10Ta-1.6Zr (wt%) were made by arc-melting, with and without 3 wt% Cu, heat-treated in two stages to be α + β, and thereafter quenched.

When studied at a higher magnification and using TKD, the bright-contrast areas in the TTZ-Cu-HT revealed a bi-lamellar microstructure, consisting of broad α lamellae and β transformed to fine α′ lamellae with various orientations, up to 58.2 area%. Increased magnification showed that TTZ-HT also had a small proportion (17.2%) of retained β (bright-contrast phase) in the dark-contrast areas. Both the Ta and Ta + Cu contents were higher in the bright-contrast phase and corresponded to the predicted composition of β at the heat treatment temperature for the TTZ alloy.

The hardness increased with Cu addition due to solution hardening. The TTZ-Cu-HT alloy was harder than that without copper, probably due to the bi-lamellar structure.

In order for the thermodynamic predictions to be accurate for the TTZ-Cu alloys, the Cu-Ta-Ti and Cu-Ta-Zr systems needed to be included in the database. However, the agreement between the calculated and experimental results was good for the TTZ alloy.

In the primary β (bright-contrast phase) in the TTZ-Cu-HT, the Ta content was only 14.5 wt%, while the Cu was 2.7 wt%, giving a total of 17.2 wt%, indicating that Cu reduced the solubility of Ta in the β phase. The potential good properties of the TTZ alloys and the absence of Al and V indicate the potential use as a suitable material for dental implants, although further studies to optimise the alloys are necessary.

## Figures and Tables

**Table 1:** Area fractions of phases in the bright- and dark-contrast areas.

**Alloy**	Area *	**Bright- Contrast Phase (%)**	**Dark-Contrast Phase (%)**
TTZ-HT	Dark contrast (N = 10) **	17.2 ± 2.5	82.8 ± 2.5
TTZ-Cu-HT	Dark contrast (N = 4)	21.3 ± 3.9	78.7 ± 3.9
	Bright contrast (N = 9)	58.2 ± 1.7	41.8 ± 1.7

* Magnification given in Figure 3. ** In the TTZ-HT alloy only dark-contrast areas were visible.

**Table 2:** Average compositions of each phase in the heat-treated TTZ-HT alloy in wt%.

**Element**	**Dark-Contrast Phase**	**Medium-Contrast Phase**	**Bright-Contrast Phase**
Ti	90.5±0.6	90.3±0.6	79.8±2.4
Cu	0.7±0.5	0.5±0.5	2.8±1.8
Zr	1.3±0.1	1.3±0.2	1.8±0.2
Ta	6.8±0.7	7.2±0.7	14.5±3.6
W	0.7±0.1	0.7±0.2	1.1±0.2

**Table 3:** Average compositions of each phase of the heat-treated TTZ-Cu-HT alloy in wt%.

**Element**	**Medium-Contrast Phase**	**Dark-Contrast Phase**	**Bright-Contrast Phase**
Ti	90.3±0.4	90.3±0.6	76.2±1.5
Zr	1.4±0.2	1.4±0.1	1.9±0.1
Ta	7.5±0.7	7.5±0.4	20.5±1.4
W	0.8±0.2	0.8±0.1	1.4±0.2

**Table 4:** Average compositions of each phase in the heat-treated TTZ-HT alloy in at%.

**Element**	**Medium-Contrast Phase**	**Dark-Contrast Phase**	**Bright-Contrast Phase**
Ti	96.9 ± 0.2	96.9 ± 0.1	92.3± 0.6
Zr	0.8 ± 0.1	0.8 ± 0.1	1.2 ± 0.1
Ta	2.1 ± 0.1	2.2 ± 0.0	6.6 ± 0.5
W	0.2 ± 0.0	0.2 ± 0.0	0.4 ± 0.1

**Table 5:** Average compositions of each phase in the heat-treated TTZ-Cu-HT alloy in at%.

**Element**	**Medium-Contrast Phase**	**Dark-Contrast Phase**	**Bright-Contrast Phase**
Ti	96.6 ± 0.3	96.6 ± 0.2	91.8 ± 0.9
Cu	0.6 ± 0.4	0.4 ± 0.4	2.3 ± 1.6
Zr	0.7 ± 0.1	0.7 ± 0.1	1.1 ± 0.1
Ta	1.9 ± 0.2	2.0 ± 0.2	4.4 ± 1.2
W	0.2 ± 0.0	0.2 ± 0.0	0.4 ± 0.1

## Data Availability

The original contributions presented in this study are included in the article. Further inquiries can be directed to the corresponding authors.
